# Estimating the Magnitude of Illicit Cigarette Trade in Bangladesh: Protocol for a Mixed-Methods Study

**DOI:** 10.3390/ijerph17134791

**Published:** 2020-07-03

**Authors:** S. M. Abdullah, Rumana Huque, Linda Bauld, Hana Ross, Anna Gilmore, Rijo M. John, Fiona Dobbie, Kamran Siddiqi

**Affiliations:** 1 Department of Economics, University of Dhaka, Dhaka 1000, Bangladesh; rumanah14@yahoo.com; 2 ARK Foundation, Suite C–3 & C–4, House–6, Road–109, Gulshan-2, Dhaka 1212, Bangladesh; 3 Usher Institute, Old Medical School, University of Edinburgh, Teviot Place, Edinburgh EH8 PAG, UK; Linda.Bauld@ed.ac.uk (L.B.); fiona.dobbie@ed.ac.uk (F.D.); 4 School of Economics, University of Cape Town, Private Bag, Rondebosch, Cape Town 7701, South Africa; hana.ross@uct.ac.za; 5 Department for Health, University of Bath, Claverton Down, Bath BA2 7AY, UK; A.Gilmore@bath.ac.uk; 6 Centre for Public Policy Research, Ernakulam, Kerala 682020, India; rmjohn@gmail.com; 7 Department of Health Sciences, University of York, Heslington, York YO10 5DD, UK; kamran.siddiqi@york.ac.uk

**Keywords:** tobacco, smoking, illicit trade, low- and middle-income countries, Bangladesh

## Abstract

The illicit tobacco trade undermines the effectiveness of tobacco tax policies; increases the availability of cheap cigarettes, which, in turn, increases tobacco use and tobacco related deaths; and causes huge revenue losses to governments. There is limited evidence on the extent of illicit tobacco trade particularly cigarettes in Bangladesh. The paper presents the protocol for a mixed-methods study to estimate the extent of illicit cigarette trade in Bangladesh. The study will address three research questions: (a) What proportion of cigarettes sold as retail are illicit? (b) What are the common types of tax avoidance and tax evasion? (c) Can pack examination from the trash recycle market be considered as a new method to assess illicit trade in comparison to that from retailers and streets? Following an observational research method, data will be collected utilizing empty cigarette packs from three sources: (a) retailers; (b) streets; and (c) trash recycle market. In addition, a structured questionnaire will be used to collect information from retailers selling cigarettes. We will select post codes as Primary Sampling Unit (PSU) using a multi-stage random sampling technique. We will randomly select eight districts from eight divisions stratified by those with land border and non-land border; and within each district, we will randomly select ten postcodes, stratified by rural (five) and urban (five) PSU to ensure maximum geographical variation, leading to a total of eighty post codes from eight districts. The analysis will report the proportions of packs that do not comply with the study definition of illicit. Independent estimates of illicit tobacco are rare in low- and middle-income countries such as Bangladesh. Findings will inform efforts by revenue authorities and others to address the effects of illicit trade and counter tobacco industry claims.

## 1. Introduction

The World Health Organization’s (WHO) Framework Convention on Tobacco Control (FCTC) suggests a number of measures to reduce the demand for and supply of tobacco. Elimination of all forms of illicit tobacco trade (Article 15) is an essential tobacco control measure [1,2]. Illicit trade may include large- and small-scale smuggling, illicit manufacturing, and counterfeiting of existing brands [1]. Cigarettes are a particularly attractive product to smugglers [2]. As cigarettes in Low- and Middle-Income Counties (LMICs) have relatively higher taxes compared to other tobacco products such as birris and smokeless tobacco [3], evading tax by diverting cigarettes into the illicit market (where sales are largely tax-free) can generate a considerable profit margin for smugglers [2,4]. Illicit trade undermines the effectiveness of tobacco tax policies, results in availability of cheap cigarettes to smokers, and thus increases tobacco use and tobacco related deaths [2,3]. Elimination or curbing illicit trade will therefore increase price and tax revenue to governments and reduce tobacco consumption and tobacco-related premature deaths [3,5].

The share of illicit in total cigarette consumption is estimated to be 11.6% globally and 16.8% in LMICs [2]. Tobacco companies claim that illicit trade in cigarettes has been growing rapidly since the 1990s [4], and often use inflated estimates of illicit cigarettes to argue against cigarette tax increases [5,6,7,8]. In the absence of national-level independent data on the extent of illicit cigarette trade and limited data on cigarette confiscation provided by the enforcement authorities [3,9], tobacco companies manipulate evidence to their advantage and use these tactics to continually lobby governments for reducing cigarette taxes [3,6,7].

In Bangladesh, 35.3% of adults (38 million) use tobacco regularly, 14% (15 million) use cigarette, and 5% (5.3 million) use “birri” [10]. However, there has been no independent published evidence measuring the extent of illicit tobacco trade in Bangladesh. Nevertheless, following industry estimates, it has been claimed that the illicit cigarette trade would be about 2% of the total cigarette market, leading to a revenue loss of 8 billion Taka, 4% of the total tobacco revenue [11]. 

This protocol will present a mixed-methods study to estimate the extent of illicit cigarette trade in Bangladesh. Three research questions will be addressed: (a) What proportion of cigarettes sold as retail in Bangladesh are illicit? (b) What are the common types of tax avoidance and tax evasion in Bangladesh? (c) Can pack examination from trash recycle market be considered as a new method to assess illicit trade in comparison to that from retailers and streets? Findings from this study will help policy makers develop more effective tobacco control measures and refute industry claims in Bangladesh. 

## 2. Materials and Methods 

Measuring illicit tobacco trade is a complex task due to the illegal nature of the activities involved [2]. Various methods have been used to assess the extent of illicit trade, such as measuring the difference between consumption and tax paid sales, interviewing smokers, studying features of cigarette packs and econometric modeling, each having their own strengths and weaknesses [3,4,12,13]. We will use observational methods and collect data from empty cigarette packs from three sources: (a) from the retailers resulting from their loose cigarette sale [3,14]; (b) littered packs from the streets surrounding the retailers [3,15,16]; and (c) littered packs from trash recycle market. The last one can be considered as an innovation in the methodology for not being applied before and could be particularly useful in LMICs where trash recycle markets are established. Regardless of the pack source, such observational studies provide a direct way to assess illicit trade [7,17].

Once collected, we will examine certain characteristics of cigarette packs such as “no mention of retail price”, “mention of brand element”, “no graphic and textual health warnings”, “no declaration”, “a duty-free sign”, and “absence of correct and authentic tax stamps” of cigarette packs as indicators of being illicit [12]. Such information could also be collected either directly from tobacco users and/or by inspecting tobacco users’ packs. However, as “loose selling” of cigarettes is common and permitted by law in Bangladesh, consumer surveys and pack inspections are of limited use. On the other hand, loose selling makes retailer pack collections more relevant. We will therefore adopt the approach developed by John and Ross (2018) [3] to measure the extent of illicit cigarette sold as retail in Bangladesh. In addition, a structured questionnaire will be used to collect information from retailers selling cigarettes.

### 2.1. Study Sites and Sampling Design

**Figure A1:**
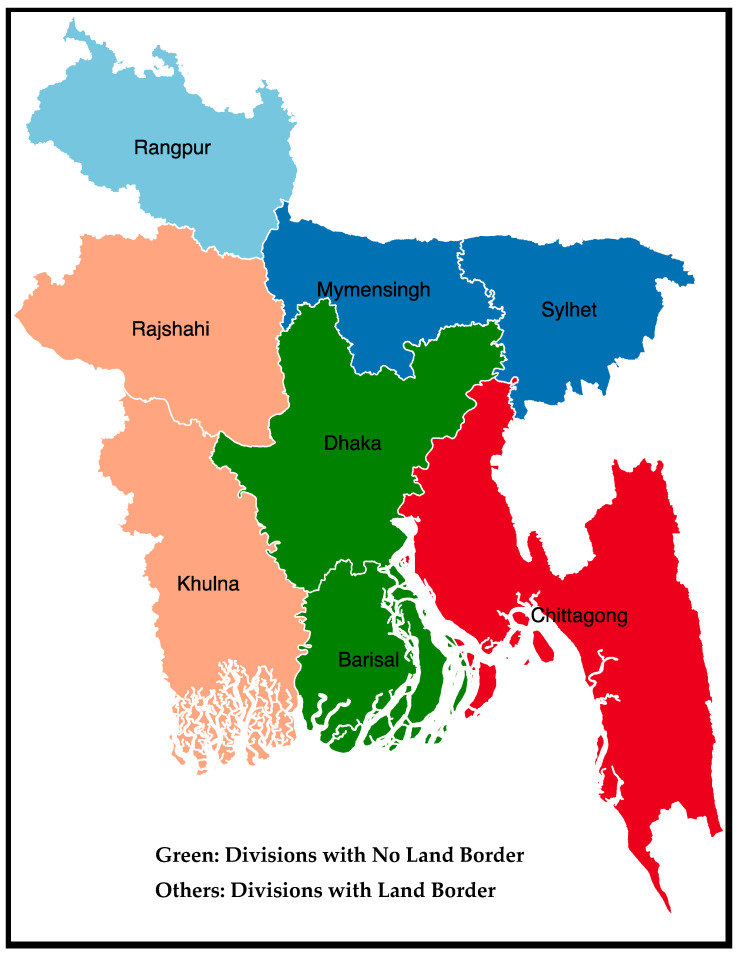
Bangladesh—divisional status with regards to border.

Bangladesh is divided into eight administrative divisions (highest administrative unit) and 64 districts. Bangladesh also has large international land borders with neighboring countries, and therefore 31 districts in six divisions border with India and Myanmar (Table 1). In Appendix A, Figure A1 presents the map of Bangladesh where the green colored places represent the two divisions with no land border area and the rest share land borders with neighboring countries. 

We will use post codes as the primary sampling unit (PSU) and select PSUs using a multi-stage random sampling technique [14]. In a division having districts with borders, we will only consider those districts having borders for inclusion in the study, and randomly select one such district from the division. For instance, while considering Rajshahi division, among eight districts, four (namely, Naogaon, Rajshahi, Nwabganj, and Joypurhat) have areas with a land border. Therefore, the sampling frame would consist of those four districts only and one will be randomly selected. In two divisions, Dhaka and Barisal—where there is no district with international land border—we will randomly select one district from each. For example, in Barishal division, there are six districts (namely, Bhola, Barishal, Barguna, Pirojpur, Patuakhali, and Jhalokathi) and none of them have international border, hence all of them would be considered in the sampling frame for the purpose of random selection. Hence, eight districts will be selected, one from each division. 

Within a district, we will select 10 post codes randomly, 60 post codes from border and 20 from non-boarder districts—a total of 80 post codes from eight districts. The PSUs in each district will also be stratified by rural (five post codes) and urban (five post codes) to ensure maximum geographical variation. Post codes (PSUs) under city corporation or paurasava (municipality) will be defined as “urban” while postcodes outside of those places will be considered as rural. Currently, there are 1216 post codes, each with an average area of 121 square kilometers. The list of the post codes is available by districts and sub districts [18]. Table 2 illustrates the sampling design. 

**Figure A2:**
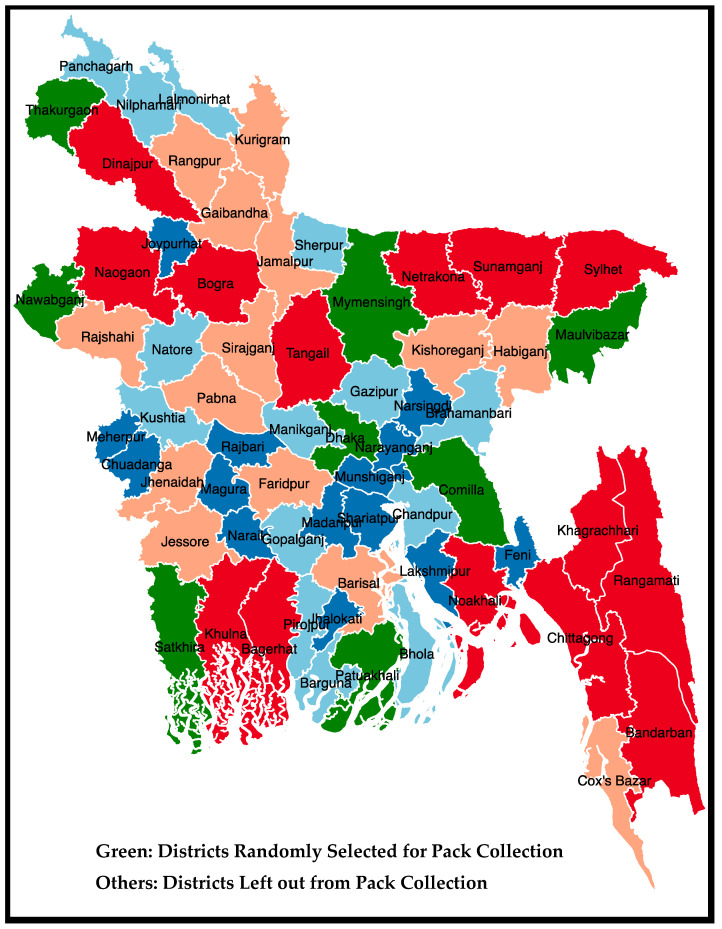
Randomly selected districts for pack collection.

We will collect the list of districts for each specific division and organize them with respect to their border status. A two-digit serial number would be assigned for each district and following randomization the respective one will be finally selected. Figure A2 in Appendix A presents the selected districts in each division covering urban–rural feature and border–non-border status from where cigarette packs would be collected. 

### 2.2. Data Collection and Management

From each PSU, three methods will be used—collecting packs from: tobacco retailers; those discarded in the street; and those discarded in trash recycle markets. An interview of the same cigarette retailers supplying packs for the study will also be carried out. 

#### 2.2.1. Collecting Packs from Tobacco Retailers

The data collection team will determine a central point (such as a bus station, a government building, or a market place) in each PSU. All types of shops, such as tea stalls, kiosks, supermarkets, department stores, and cigarette shops that sell cigarettes or birri, or both, would be eligible to take part in the study. Enumerators will walk half a kilometer in both directions on the main street around the public gathering place and approach all the retailers meeting the eligibility criteria. All eligible retailers will be provided with both verbal and written information about the study. If they agree to take part, they will be required to sign the consent form. Following consent, the field worker will proceed to pack collection. The retailers would be supplied with a “collection bag” on the previous day and asked to keep all the empty packs resulted from loose sale on the following day since the shop opened in that bag. All “collection bags” will be retrieved by the field workers at the end of the business day. We will provide a small monetary reward of BDT 3 (GBP 0.03) per empty pack deposited in the collection bag. In case the empty pack of the cheapest and popular brands were not available in the bag from a particular retailer, the team would take a picture and code all the relevant attributes of such pack observed in the shop itself [3]. 

#### 2.2.2. Collecting Discarded Packs from Streets

In addition to collecting empty packs from the tobacco retailers, discarded packs from the streets where those retailers are established and also from some other locally important streets around public gathering places will be accumulated [3]. The distance that would be covered in each street would be one kilometer (half a kilometer in each direction). The field workers would use separate “collection bag” with unique identification number for collecting the discarded packs in each area. The pack collection from streets would take place simultaneously during the day when the “collection bag” to the retailers would be distributed. The street selection would also consider the street condition for walking and possibility of finding littered packs. Necessary safety equipment such as masks and gloves will be provided to the field staff while collecting littered packs. 

#### 2.2.3. Collecting Discarded Packs from Trash Recycle Markets

A common practice in Bangladesh is to recycle waste papers, including cigarette packs, which are then used as raw material in paper mills. Large municipal cities in urban areas locally known as “City Corporations” have a trash collection system. They collect trash from confined areas and from street corners where the trash bins are placed. A trash recycle market exists in some places to collect used papers and cigarette packs, but this is limited in rural areas. If a trash recycle market exists in the sample areas, and we are able to obtain the empty packs, we will include them in the study. 

#### 2.2.4. Interview of Point of Sale Retailers

During bag collection, basic information such as name and type of the store, price and estimated quantity of the cheapest brand sold in the shop, name and price of most popular brand, and the estimated quantity sold will be collected. In addition, they will be asked about the profit share of tobacco products in their total daily profit, sources of supply, incentives from the suppliers’ end, market supervision, and knowledge, understanding, and perception regarding illicit tobacco products. A structured questionnaire that has been used in studies in the UK, Bangladesh, Nepal, and Pakistan previously will be adapted to collect these data from the retailers [19]. Following the Tobacco Advertisement and Promotion Survey (TAPS) [20], a standard pack analysis tool has been developed. 

#### 2.2.5. Field Design and Implementation

Ten data collectors will be recruited and attend a one-day training session on: (a) getting packs from retailers; (b) getting packs from the streets; and (c) collecting packs from trash recycle market. The in-house training will focus on the ways of finding the public gathering places, selecting the retailers, instructing the retailers about pack preservation for the day maintaining certain qualities, and collecting the packs. They will also receive training on obtaining informed and written consent, how to generate the “Unique ID” for the “Collection Bags”, how the packs should be preserved, and how to collect discarded packs from the streets. The data collection would be carried out during the period February 2020 to September 2020 and the analyses be performed by March 2021.

The Research Fellow and one field supervisor will visit the study sites during data collection period to check the survey is done properly. They will also visit a subset of the chosen retailers. Data will be monitored for quality and completeness by the core research team. Due to the sensitive nature of the research study, we will make our best efforts to provide security measures to our data collectors. We will group the data collectors together, whenever possible. We will prescribe specific hours for data collection, outside of which data collectors will not be allowed to collect data. Every data collector will have a cell phone with pre-paid credit and a dedicated team member will be on-call during the prescribed data collection time, to provide assistance regarding the questions or any issue in the field. 

All the collection bags will be brought back to the research office where we will have data entry operators to inspect the packs and register their characteristics into the database for further analysis. The training for data entry operators will take place with the direct support from National Board of Revenue (NBR) officials in Bangladesh. The relevant officials will be invited to share the techniques and expertise they employ when they examine the authenticity of tax stamps on cigarettes. 

#### 2.2.6. Ethical Considerations

The primary principles of good ethical practice such as autonomy, justice, beneficence, and non-maleficence will be maintained during the study. We are conscious that retailers could have concerns that the research may negatively impact their business, and interviewers could feel uncomfortable during the interaction with retailers. We will adopt multiple measures to mitigate these concerns. First, all potential retailers will be provided with information regarding the project and written consent will be taken prior to data collection. Those who do not agree to participate after reviewing the study information will not be enrolled in the study. The reasons for declining to participate in the study will be recorded. Second, retailers will be reassured that all information collected during the course of the study will be kept strictly confidential. Third, researchers will be trained on techniques for handling conflict, threats, abuse, or compromising situations. We will specifically train researchers to ask questions in a non-judgmental manner and not to put any pressure on the respondents, if they show signs of reluctance in answering one or more questions. Our data collection team will be respectful to the retailers and will avoid interference with the normal flow of business. Interviewers will only continue the interview when the retailer is not dealing or in the vicinity of customers. Every interviewer will have the right to decline collecting packs from a retailer in a location that makes him or her feel unsafe. 

The primary principles of good ethical practice such as autonomy, justice, beneficence, and non-maleficence will be maintained during the study. We are conscious that retailers could have concerns that the research may negatively impact their business, and interviewers could feel uncomfortable during the interaction with retailers. We will adopt multiple measures to mitigate these concerns. First, all potential retailers will be provided with information regarding the project and written consent will be taken prior to data collection. Those who do not agree to participate after reviewing the study information will not be enrolled in the study. The reasons for declining to participate in the study will be recorded. Second, retailers will be reassured that all information collected during the course of the study will be kept strictly confidential. Third, researchers will be trained on techniques for handling conflict, threats, abuse, or compromising situations. We will specifically train researchers to ask questions in a non-judgmental manner and not to put any pressure on the respondents, if they show signs of reluctance in answering one or more questions. Our data collection team will be respectful to the retailers and will avoid interference with the normal flow of business. Interviewers will only continue the interview when the retailer is not dealing or in the vicinity of customers. Every interviewer will have the right to decline collecting packs from a retailer in a location that makes him or her feel unsafe. 

The study has been granted ethics approval by the National Research Ethics Committee of the Bangladesh Medical Research Council (Ref: BMRC/NREC/2016–2019/344; Registration Number: 241 05 08 2019) and the Health Sciences Research Governance Committee at the University of York (HSRGC/2019/346/A, approval date 5 July 2019). 

## 3. Analysis Plan

We will classify a cigarette pack as illicit if it has at least one of the following attributes:

1. No mention of “retail price”: It is mandatory to print the retail price of the goods “on the body of the goods or on every package, sachets or cells distinctly, conspicuously and indelibly”. This applies to cigarettes, and packs not containing the retail price can be considered illegal [21].
2. Mention of brand element: Packets should not contain any brand elements, for instance Light, Mild, Low Tar, Extra, and Ultra. Packs mentioning brand element will be considered illegal [22].
3. No graphic health warnings: Health warnings shall be printed on both sides of the packet covering at least 50% of the total area of each main display area. In addition, the warnings should be in colored pictures. Rules notified further suggests that the ratio and written warnings should be 6:1 and the written warnings has to be printed in black background with white font color. Packs not fully complying the above rules will be considered illegal [21,22].
4. No textual health warnings: The following warnings should be printed on the packet in Bengali, otherwise it can be considered as illegal [21,22].
  (a) Smoking causes throat and lung cancer;
  (b) Smoking causes respiratory problems;
  (c) Smoking causes stroke;
  (d) Smoking causes heart disease;
  (e) Second-hand smoke causes harms to the fetus;
  (f) Smoking causes harms to the fetus; or
  (g) Second-hand smoking causes death.
5. No declaration: Selling of tobacco products without having the statement “Sales allowed only in Bangladesh” printed on the packets would be illegal [22].
6. A duty-free sign: Cigarette packs collected from retailers with a “duty-free” sign will be classified as illegal as these are to be sold in Duty Free shops only and should not be available in the market.
7. Absence of correct and authentic tax stamp/banderole: The government has made it obligatory to use tax stamp or banderole on cigarette packets. Any sale of cigarette without this tax stamp or banderole is legally prohibited. The banderole/stamp of cigarette pack of different size and design had been defined [21]. We will check if the packs have used banderole/stamp correctly as per the SRO and VAT Booklet, and if the banderole/stamp is genuine.

In general, all pack characteristics considered for detecting legitimacy in the retailer sample might also be accepted for those in littered or trash samples. Nevertheless, one of the limitations of the above general approach is that it might fail to distinguish between tax evasion and avoidance. More specifically, there may be some legally purchased duty-free packs as well as those bought from neighboring countries, in a sample from street, while also having illegally purchased and illegally sold packs in the retail shops belonging to that street. This possibility might result in under- or overestimation of the illicit cigarette trade. Hence, it rationalizes the application of an adjustment factor while measuring the magnitude of illicit cigarette in the sample collected particularly from street and trash recycle market. Table 3 exemplifies a thematic presentation of legitimacy criterion of a cigarette pack and adjustment requirement in the estimation. 

Assuming that the littered packs in the streets would by and large reflect the average characteristics of those sold by different retailers on the same street, the following adjustment method would be applied:

Suppose DF_L_ is the proportion of duty-free packs (both legal and illegal) in the littered sample and DF_R_ is the proportion of duty-free packs (all illegal) in retailer sample. Then, the estimated proportion of illegal duty-free packs from littered sample would be

=DF_R_ if DF_L_ > DF_R_; and

=DF_L_ if DF_L_ ≤ DF_R_.  

Similar adjustment process would be followed while estimating extent of illicit cigarette using packs from trash recycle sample. It should be noted here that self-made cigarette using tax-paid tobacco powder or crushed tobacco may not be the scope of current work. However, a pack for a local category of cheap cigarette known as “birri” would be collected and examined for its compliance as per the study definition. Statistical Analysis (STATA) software version 15 would be used. The main statistical analysis will report the frequencies and proportions of cigarette packs that do not comply with the tobacco control laws and falls within the study definition of illicit. 

The packs collected from different sources would be kept in separate bags and analyzed separately. In addition, the features of the respective packs would be recorded in three different databases to find separate estimates of illicit cigarettes. The estimates from first two inter-related methods will be triangulated and hence complementing each other. We will then compare and validate these findings with the third method. Given the availability of the trash market in the study sites, and adequate volume of packs collected from the trash market, the findings will be compared with the first two methods. We will assess the extent to which the result of trash market measure corresponds to first two measures to estimate of illicit trade for its validity. Findings from pack analysis would be further triangulated with the retailer interview findings for better understanding of the pattern of illicit practices in the cigarette market. 

## 4. Discussion

Over the last two and half decades, several significant advances have been made on studying illicit tobacco trade. This body of evidence has identified the following three key themes of particular relevance for research on illicit tobacco trade: (1) different ways to measure illicit tobacco trade; (2) distortion of the data by tobacco industry; and (3) effectiveness of interventions to address the problem of illicit trade [23]. 

The empirical estimation of the extent of illicit trade in the tobacco market often remains an intricate and challenging task. Tobacco control researchers around the world often triangulate several methods to improve the precision of their estimates of the magnitude of illicit trade [24]. The volume of scientific literature is skewed towards the high- and upper middle-income countries [1,4,5,6,7,17,25,26,27,28,29,30,31,32,33,34] relative to the low- and lower middle-income countries [11,24,35,36,37,38]. This is perhaps due to the issue of data availability and research capacity. Compared to LMICs, a greater potential loss in government revenue due to illicit trade in high-income countries, owing to higher tax rates, results in a higher public investment on related research. On the other hand, the dimension of illicit trade in LMICs is different [1]. Although the extent of financial incentive would be lower for illicit tobacco traders in those countries, the risks associated with such malicious activities would also be lower due to lack of governance, which might encourage the illicit traders to extend their activities there. Therefore, the importance of research in illicit trade in Bangladesh cannot be ignored. 

In addition, existing works prescribed to follow country specific approach towards illicit trade as uniform ranking of research priorities within the arena appears to be impossible [1,34]. Taking into account the country context, this attempt would be a considerable contribution to the field of illicit tobacco trade research in Bangladesh.

This study is being conducted as part of a research consortium of fifteen partner organizations across eight countries (Bangladesh, Ethiopia, Ghana, India, South Africa, the Gambia, Uganda, and the UK) funded by the Global Challenges Research Fund in the UK and named the “Tobacco Control Capacity Programme” [39]. We have established a national stakeholders’ group in Bangladesh and arranged several stakeholder engagement meetings to get their feedback on the study protocol. 

The successful implementation of the protocol is subject to endorsed inspection of collected packs and data recording about the authenticity of tax stamp. According to the local tobacco control laws, a tax paid pack must contain the tax stamp and we are in need of examining the security features of the tax stamp for study purpose. In practice, in most cases, these tax stamps are affixed in the upper left- or right-hand corner in the outer part of a cigarette pack. Thus, it might happen that the required tax stamp is removed, lost, or torn while opening by the retailers or littering by the smokers into the streets and trash bins. Considering this potential threat, the gold standard would have been collecting the unopened packs for tax and other compliance inspection [12]. This scope is limited in the current context due to the fund challenge that has to be made with respect to the sampling design. Hence, training on eligibility criterion for an empty cigarette pack to be included in the inspection basket would be of sheer importance. All the packs where tax stamps are not in proper status to be checked for authenticity would be excluded from the study. 

Another important potential limitation of the proposed methodology would be its ability of correct estimation for the size of illicit market. Since the unit of observation from where packs would be collected is retail cigarette shops, selection bias might remain present in the estimation. The estimate would be overestimated if pack collection takes place in an area where illegal tobacco products are known to be traded. In contrast, the estimates would be underestimated if retailers purposefully preserve the licit cigarette packs in the collection bag. Moreover, conditioning that illicit cigarettes are sold in full packs in general rather than single sticks, collecting empty packs from retailers might underestimate the magnitude. Considering all these potential threats, we expect the study would be free from selection bias as the retailer selection would be completely random covering the urban–rural and border–non-border perspective around the country. The validity and representativeness of the estimate measured using the packs collected from retailers would be ensured by comparing that with the estimate retrieved from littered packs from the streets and packs collected from the trash recycle market in the same neighborhood. Estimates from the two later sources to a greater extent would address the limitations if retailer deposit only licit packs or illegal cigarette happens to be sold in full pack. The comparison of results and further analysis of illicit packs from these three sources would help us to examine the existence of any systematic difference among them. Absence of systematic difference would ensure the possible unbiasedness of the estimates [12].

Finally, in the absence of any prevalence data at the division or district level, it would not be possible to propose a sample size weighted on the local smoking prevalence or smoking intensity. Bangladesh has completed two rounds of Global Adult Tobacco Survey (GATS). However, the first round does not have district level or divisional level information on smoking prevalence while the data from second round have not been released yet. 

Contemplating the context of Bangladesh, the data challenges along with the data quality required in other methods increases the suitability of observational analysis. In addition, it would help researchers and in turn policy makers to learn about the pattern of illicit activities related to packaging compliance and initiate the proper policy measure henceforth. In addition, the estimate along with analysis would help us to enhance our knowledge regarding how an important stakeholder in supply chain (retailers) is legally used to reach to the smokers. 

## 5. Conclusions

In this paper, we describe a protocol for a mixed-methods research study to estimate the extent of illicit cigarette sold as retail in Bangladesh. This research aims to better understand the proportion of illicit cigarettes traded in the retail market, as well as the common types of tax avoidance and tax evasion in the country. It will also give effort to validate a new method to assess illicit cigarette trade with the established methods. The study will potentially be of interest to a wider audience, including but not limited to, academia, government and non-government organizations, and tobacco control and policy implementation researchers, particularly because independent estimates of illicit tobacco are rare in LMICs such as Bangladesh. Moreover, findings will inform efforts to address the effects of illicit trade and counter tobacco industry claims. The strength of this research particularly relies on two aspects; one is the methodology design that has been successfully applied in a country with similar characteristics and the other one is robust sampling design covering urban–rural and border–non-border geographical areas across the country. 

While estimating the magnitude of illicit trade there is established evidence of using packs directly collected from smokers. Deliberating the context and required study design, we believe that might be a future research option in the field of illicit trade research in Bangladesh. Moreover, effectiveness of different intervention tool put forwarded for controlling illicit trade and consequently the revenue loss of the country would be another important perspective of future research.

## Figures and Tables

**Table 1:** Administrative areas with border status and sampling frame.

Border Areas	Division	Districts with Land Border	Districts without Land Border	Sampling Frame for District Selection
Yes	Sylhet	4	0	4 Bordering Districts
	Rangpur	6	2	6 Bordering Districts
	Rajshahi	4	4	4 Bordering Districts
	Mymensingh	4	0	All Districts
	Khulna	6	4	6 Bordering Districts
	Chittagong	7	4	7 Bordering Districts
	Total	31	14	
No	Dhaka	0	13	All Districts
	Barishal	0	6	All Districts
Country Total		31	33	

Source: Population Census, 2011.

**Table 2:** Sampling Design.

Boarder Status	No Land Border Area		Have Land Border Area						Area Design and Selection Method
**Stage - One (Division)**									
**8 Divisions**	**DIV - 1**	**DIV - 2**	**DIV - 3**	**DIV - 4**	DIV - 5	DIV - 6	DIV - 7	DIV - 8	All Divisions Covered (No Randomization)
**Stage - Two (District)**									
8 Districts	DIS - 1	DIS - 2	DIS - 3	DIS - 4	DIS - 5	DIS - 6	DIS - 7	DIS - 8	**No Land Border Area:** Randomly Selected from the List of District who have No Land Border **Have Land Border Area:** Randomly Selected from the List of Districts from a Division who have Land Border Area.
**Stage - Three (Sub - District)**									
5 UPCs form Each District Covering City Corporation/Metropolitan Area/Municipality Area (Paurasava)	UPC - 1	UPC - 1	UPC - 1	UPC - 1	UPC - 1	UPC - 1	UPC - 1	UPC - 1	**Urban Post Codes (UPC):** In each district UPC would be those areas (Sub - Districts) covering Metropolitan Cities or City Corporation Areas or Municipality Area (Paurasava) **Selection Method:** Simple Random Sampling
	UPC - 2	UPC - 2	UPC - 2	UPC - 2	UPC - 2	UPC - 2	UPC - 2	UPC - 2	
	UPC - 3	UPC - 3	UPC - 3	UPC - 3	UPC - 3	UPC - 3	UPC - 3	UPC - 3	
	UPC - 4	UPC - 4	UPC - 4	UPC - 4	UPC - 4	UPC - 4	UPC - 4	UPC - 4	
	UPC - 5	UPC - 5	UPC - 5	UPC - 5	UPC - 5	UPC - 5	UPC - 5	UPC - 5	
5 RPCs/BPCs form Each District Situated in different Sub Districts (Outside City Corporation/ Metropolitan Area/Municipality Area)	RPC - 1	RPC - 1	BPC - 1	BPC - 1	BPC - 1	BPC - 1	BPC - 1	BPC - 1	**Rural Post Codes/Border Area Post Codes (RPC/BPC):** **No Land Border Area:** RPC would be those areas (Sub - Districts) covering outside Metropolitan Cities or City Corporation Areas or Municipality areas (Paurasava). **Have Land Border Area:** BPC would be those areas (Sub - Districts) covering outside Metropolitan City or City Corporation Areas Municipality areas (Paurasava) as well as sharing land border. **Selection Method:** Simple Random Sampling
	RPC - 2	RPC - 2	BPC - 2	BPC - 2	BPC - 2	BPC - 2	BPC - 2	BPC - 2	
	RPC - 3	RPC - 3	BPC - 3	BPC - 3	BPC - 3	BPC - 3	BPC - 3	BPC - 3	
	RPC - 4	RPC - 4	BPC - 4	BPC - 4	BPC - 4	BPC - 4	BPC - 4	BPC - 4	
	RPC - 5	RPC - 5	BPC - 5	BPC - 5	BPC - 5	BPC - 5	BPC - 5	BPC - 5	

**Table 3:** Legitimacy Criterion of Cigarette Pack and Bias Correction.

Pack Source	Tax Status	Packaging Compliance	Legitimacy of Cigarette Pack	Bias Correction in Estimate
**Retailers**	Paid	Compliant	Legal	No Adjustment Required
		Not Complaint	Illegal	
	Not Paid	Compliant	Illegal	
		Not Complaint	Illegal	
**Streets/Trash Recycle Market**	Paid	Compliant	Legal	No Adjustment Required
		Not Complaint	Might be Legal or Illegal	Adjustment Applied
	Not Paid	Compliant	Might be Legal or Illegal	
		Not Complaint	Might be Legal or Illegal	

## References

[1] Van Walbeek C., Blecher E., Gilmore A., Ross H. (2012). Price and tax measures and illicit trade in the framework convention on tobacco control: What we know and what research is required. Nicotine Tob. Res..

[2] Joossens L., Merriman D., Ross H., Raw M. (2010). The impact of eliminating the global illicit cigarette trade on health and revenue. Addiction.

[3] John R.M., Ross H. (2018). Illicit cigarette sales in Indian cities: Findings from a retail survey. Tob. Control.

[4] Van Walbeek C. (2014). Measuring changes in the illicit cigarette market using government revenue data: The example of South Africa. Tob. Control.

[5] Joossens L., Lugo A., La Vecchia C., Gilmore A.B., Clancy L., Gallus S. (2014). Illicit cigarettes and hand-rolled tobacco in 18 European countries: A cross-sectional survey. Tob. Control.

[6] Gilmore A.B., Rowell A., Gallus S., Lugo A., Joossens L., Sims M. (2014). Towards a greater understanding of the illicit tobacco trade in Europe: A review of the PMI funded ‘Project Star’ report. Tob. Control.

[7] Stoklosa M., Ross H. (2014). Contrasting academic and tobacco industry estimates of illicit cigarette trade: Evidence from Warsaw, Poland. Tob. Control..

[8] Gallagher A.W., Evans-Reeves K.A., Hatchard J.L., Gilmore A.B. (2019). Tobacco industry data on illicit tobacco trade: A systematic review of existing assessments. Tob. Control.

[9] Chen J., McGhee S.M., Townsend J., Lam T.H., Hedley A.J. (2015). Did the tobacco industry inflate estimates of illicit cigarette consumption in Asia? An empirical analysis. Tob. Control.

[10] World Health Organisation (WHO) (2017). Global Adult Tobacco Survey (GATS) Factsheet. http://www.searo.who.int/bangladesh/gatsbangladesh/en/.

[11] Sadiq A., Zaidi S., Khurshid A. (2019). Bangladesh-Illicit Tobacco Trade, Confronting Illicit Tobacco Trade: A Global Review of Country Experiences.

[12] Ross H. (2015). Understanding and Measuring Cigarette Tax Avoidance and Evasion: A Methodological Guide.

[13] David M. (2013). Economics of Tobacco Toolkit, Tool 7: Understand, Measure, and Combat Tobacco Smuggling.

[14] Scollo M., Bayly M., Wakefield M. (2014). Availability of illicit tobacco in small retail outlets before and after the implementation of Australian plain packaging legislation. Tob. Control.

[15] Merriman D. (2010). The Micro-Geography of Tax Avoidance: Evidence from Littered Cigarette Packs in Chicago. Am. Econ. J. Econ. Policy.

[16] Chernick H., Merriman D. (2013). Using Littered Pack Data to Estimate Cigarette Tax Avoidance in NYC. Natl. Tax J..

[17] Joossens L., Raw M. (2008). Progress in combating cigarette smuggling: Controlling the supply chain. Tob. Control.

[18] Directorate of Posts Government of the People’s Republic of Bangladesh. http://www.bangladeshpost.gov.bd/postcode/index/1.

[19] Siddiqi K., Scammell K., Huque R., Khan A., Baral S., Ali S., Watt I. (2016). Smokeless Tobacco Supply Chain in South Asia: A Comparative Analysis Using the WHO Framework Convention on Tobacco Control. Nicotine Tob. Res..

[20] Feighery E., Cohen J., Grant A., Khan A., Latif E. (2013). Assessing Compliance with Tobacco Advertising, Promotion, and Sponsorship (TAPS) Bans: A ‘How-to’Guide for Conducting Compliance Studies of Point of Sales Advertising & Product Display, Outdoor Advertising & Product Packaging.

[21] National Board of Revenue (NBR) Value Added Tax (VAT) Act 1991, Statutory Regulatory Order (SRO) [SRO–181/2011/694–VAT, SRO-182/2011/695–VAT, SRO-206/2011/619–VAT, SRO-164/2017/07–VAT, SRO-165/2017/08–VAT and SRO-208/2018/811–VAT], and VAT Booklet-11 on “Use of Stamp and Banderole” system. http://nbr.gov.bd/regulations/acts/vat-acts/eng.

[22] National Tobacco Control Cell (NTCC) Health Services Division, Ministry of Health and Family Welfare, Government of the People’s Republic of Bangladesh. https://ntcc.gov.bd/page/act-rules.

[23] Chaloupka F.J. (2014). Taxes, prices and illicit trade: The need for sound evidence. Tob. Control.

[24] Ahsan A., Wiyono N.H., Setyonaluri D., Denniston R., So A.D. (2014). Illicit cigarette consumption and government revenue loss in Indonesia. Glob. Health.

[25] Wherry A.E., McCray C.A., Adedeji-Fajobi T.I., Sibiya X., Ucko P., Lebina L., Golub J.E., Cohen J.E., Martinson N.A. (2014). A comparative assessment of the price, brands and pack characteristics of illicitly traded cigarettes in five cities and towns in South Africa. BMJ Open.

[26] Kaplan B., Navas-Acien A., Cohen E.J. (2017). The prevalence of illicit cigarette consumption and related factors in Turkey. Tob. Control.

[27] Blecher E., Liber A., Ross H., Birckmayer J. (2013). Euromonitor data on the illicit trade in cigarettes. Tob. Control.

[28] Guthrie J., Hoek J., Darroch E., Wood Z. (2015). A qualitative analysis of New Zealand retailers’ responses to standardised packaging legislation and tobacco industry opposition. BMJ Open.

[29] Maldonado N., Llorente B., Iglesias R.M., Escobar D. (2018). Measuring illicit cigarette trade in Colombia. Tob. Control.

[30] Paraje G. (2018). Illicit Cigarette Trade in Five South American Countries: A Gap Analysis for Argentina, Brazil, Chile, Colombia, and Peru. Nicotine Tob. Res..

[31] Lencucha R., Callard C. (2011). Lost revenue estimates from the illicit trade of cigarettes: A 12-country analysis. Tob. Control.

[32] Iglesias R.M., Szklo A.S., De Souza M.C., De Almeida L.M. (2016). Estimating the size of illicit tobacco consumption in Brazil: Findings from the global adult tobacco survey. Tob. Control.

[33] Arevalo R., Corral E.J., Monzon D., Yoon M., Barnoya J. (2016). Characteristics of illegal and legal cigarette packs sold in Guatemala. Glob. Health.

[34] Ross H., Husain M.J., Kostova D., Xu X., Edwards S.M., Chaloupka F.J., Ahluwalia I.B. (2015). Approaches for Controlling Illicit Tobacco Trade—Nine Countries and the European Union. MMWR Morb. Mortal. Wkly. Rep..

[35] Nguyen M.T., Denniston R., Nguyen H.T.T., Hoang T.A., Ross H., So A.D. (2014). The Empirical Analysis of Cigarette Tax Avoidance and Illicit Trade in Vietnam, 1998-2010. PLoS ONE.

[36] Nguyen M.T., Son D.T., Nguyen N.Q., Bowling M., Ross H., So A.D. (2019). Illicit Cigarette Consumption and Government Revenue Loss in Vietnam: Evidence from a Primary Data Approach. Int. J. Environ. Res. Public Health.

[37] Abola V., Sy D., Deniston R., So A. (2014). Empirical Measurement of Illicit Tobacco Trade in the Philippines. Philipp. Rev. Econ..

[38] Brown J., Welding K., Cohen J.E., Cherukupalli R., Washington C., Ferguson J., Smith K.C. (2017). An analysis of purchase price of legal and illicit cigarettes in urban retail environments in 14 low- and middle-income countries. Addiction.

[39] Dobbie F., Mdege N.D., Davidson F., Siddiqi K., Collin J., Huque R., Owusudabo E., van Walbeek C., Bauld L. (2019). Building capacity for applied research to reduce tobacco-related harm in low- and middle-income countries: The Tobacco Control Capacity Programme (TCCP). J. Glob. Health Rep..

